# SPV: a JavaScript Signaling Pathway Visualizer

**DOI:** 10.1093/bioinformatics/bty188

**Published:** 2018-03-24

**Authors:** Alberto Calderone, Gianni Cesareni

**Affiliations:** Bioinformatics and Computational Biology Unit, Department of Biology, University of Rome ‘Tor Vergata’, Rome, Italy

## Abstract

**Summary:**

The visualization of molecular interactions annotated in web resources is useful to offer to users such information in a clear intuitive layout. These interactions are frequently represented as binary interactions that are laid out in free space where, different entities, cellular compartments and interaction types are hardly distinguishable. Signaling Pathway Visualizer is a free open source JavaScript library, which offers a series of pre-defined elements, compartments and interaction types meant to facilitate the representation of signaling pathways consisting of causal interactions without neglecting simple protein–protein interaction networks.

**Availability and implementation:**

Freely available under Apache version 2 license; Source code: https://github.com/Sinnefa/SPV_Signaling_Pathway_Visualizer_v1.0. Language: JavaScript; Web technology: Scalable Vector Graphics; Libraries: D3.js.

## 1 Introduction

Signaling pathways are the mental abstraction used by biologists to represent a coordinated cascade of events leading to the expression of one or more genes ultimately resulting in observable phenotypes. These series of events are usually described as a collection of entities, proteins, small molecules, protein complexes, chemicals and so on connected by oriented or non-oriented links, representing interactions or signal propagations.

One way of representing biological pathways is the reaction-based model. In this representation, a reaction is defined by the stoichiometry of the participating elements and by differential equations specifying their dynamics. This representation is adopted, for instance, by Reactome (Fabregat *et al.*, 2016), a web resource that uses a hierarchical reaction based visualization, and by Pathway Commons (Cerami *et al.*, 2011), which also offers reaction-based models collected from external resources.

A different way of representing biological pathways is by based on causal interaction where an entity has a binary effect (activation/inhibition) on its target. Some online resources have started collecting signaling interaction information from the literature and to represent it as a set of causal relationships (Fazekas *et al.*, 2013; Perfetto *et al.*, 2015). Causal interactions are a relatively new focus of data curation and, as such, a clear automatic graphic representation is still missing.

A number of web applications have been developed to represent protein interaction network including but not limited to those based on Cytoscape.js (Franz *et al.*, 2015) such as CerebralWeb by InnateDB (Breuer *et al.*, 2013) or the visualizer of the CellWhere database (Zhu *et al.*, 2015). Other web applications are based on a different JavaScript library called D3.js (Bostock *et al.*, 2011), for instance, CellNetVis (Heberle *et al.*, 2017). CerebralWeb, CellNetVis and CellWhere offer the possibility of visualizing protein interactions within cellular compartment but they do not provide out-of-the-box graphical elements specifically designed for signaling cascades consisting of causal interactions.

Here, we introduce Signaling Pathway Visualizer (SPV) whose development started with *Mentha* (Calderone *et al.*, 2013) which stores protein–protein interaction information and continued with SIGNOR (Perfetto *et al.*, 2015) which collects causal signaling information. SPV is a JavaScript library which, by offering several already defined entities and interaction types, aims at simplifying the visualization of signaling pathways represented as a set of causal interactions in a biologically oriented intuitive top-down representation without neglecting simple protein–protein interaction networks.

## 2 Implementation

A clear, intuitive representation of signaling pathways can be hard because of two major factors: (i) the large variety of entity types and signaling effects and (ii) the need to visualize pathways in the way biologists are used to, i.e. in a top-down cascade where the signal is propagated from the membrane to the nucleus. To simplify these two tasks, we have developed a JavaScript library based on D3.js, which simplifies signaling pathways visualization. The advantages are particularly evident when representing the highly detailed annotation of SIGNOR (Perfetto *et al.*, 2015) with its variety of interacting entities, which include protein complexes, protein families, small molecules and so on, and as several different causal interaction types.

**Fig. 1.**
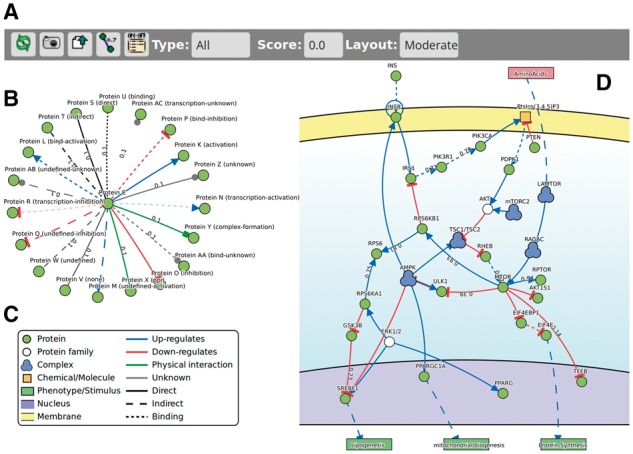
Toolbar, graphical elements supported in SPV and the mTOR pathway. The top menu bar various filtering and visualization options. SVP comes with several elements already defined such as proteins, external non-human/viral proteins, chemicals, complexes and others (**C**). Interactions can be of several types with different line strokes and one transparency effect to distinguish activations from, for instance, indirect activations, transcriptional activation etc. (**B**). All these elements can be combined to represent a signaling pathway from cellular membrane, in yellow, to nucleus, lilac (**D**) as well as simple protein-protein interaction networks (B). Protein forming a group, for instance, a complex, are enveloped in a hull which turns red on mouse hover (D)

SPV is a JavaScript library conceived to visualize signaling pathways and protein interactions by displaying entities of different types connected by different links. The library offers two distinct layouts, one for protein–protein interaction networks (Fig. 1B) and one specifically designed for causal relationships (Fig. 1D). SPV consists of a basic toolbar (Fig. 1A) and a graphical stage where the network is visualized.

We defined a collection of eight different elements and a variety of link types that can be used in any combination independent of the visualization used (Fig. 1B and C). This collection of elements and interactions can be extended by the programmer as needed. Nodes and edges are sensitive to mouse clicks which open pop-up windows displaying information about the selected elements.

Entities are represented as circles and are differently colored according to the types specified by the programmer. Complexes, phenotypes and small molecules are an exception and have distinctive shapes. We also defined a graphical representations for a variety of edge types: activation, inhibition, binding, protein-protein interaction, complex formation and others, which can have different line strokes and one transparency level. These collections of different lines can be used to represent subclasses such as ‘indirectly activates’ or ‘transcriptional activation’. Lastly, in order to represent proteins belonging to a common group, for instance, protein complexes, it is possible to envelop with gray hulls nodes belonging to user defined lists of entities, which, on mouse hover, turn red showing the group name. Protein complexes visualization goes beyond the purpose of SVP due to the intrinsic complexity of such biological entities for which a dedicated JavaScript visualizer called ComplexViewer, a visualization tool for Complex Portal (Meldal *et al.*, 2015), was recently released (Combe *et al.*, 2017).

For the visualization of signaling pathways, we define four layers representing three compartments: extracellular, cellular membrane (receptors), nucleus (transcription factors) and a bottom layer to place phenotypes. Unconstrained nodes are confined in a bounding box that represents the cytosol of the cell. This bounding box prevents nodes from going outside of the membrane and inside the nucleus but, at the same time, it lets unfixed nodes free to move according to the default layout based on spring layout (Fruchterman and Reingold, 1991) with some fine tuning, such as automatic links curvature and automatic labels placement to avoid overlaps. On the other hand, for the visualization of protein–protein interaction networks, we let all nodes move freely according to the classical spring layout.

The top menu bar offers some filtering options to visualize only specific interaction types or only connections above a certain edge score threshold so that the user can easily see if and how two nodes are connected, for instance, only by up-regulations. In addition, it offers the possibility of exporting the currently visualized image as a standard SVG to be used in publications, presentations and posters. Finally, it allows the user to export the network as plain-text comma-separated file.

## 3 Compatibility

Our group is an active member of the IMEx Consortium (Orchard *et al.*, 2012) focused on the curation of published molecular interaction extracted from peer reviewed literature. The IMEx Consortium promotes the Proteomics Standards Initiative-Molecular Interaction (PSI-MI) in order to facilitate data exchange (Hermjakob *et al.*, 2004). In particular, the JavaScript Object Notation format MI-JSON will soon be published (Combe *et al.*, 2017) and integrated in SPV.

Furthermore, we are actively working on defining a standard for causal interaction (manuscript in preparation), which will soon be formalized and integrated in SPV. In the meanwhile, we made SPV compatible with PSI-MI tab separated files downloadable via any PSICQUIC (Aranda *et al.*, 2011) server and with the SIGNOR Causal Interaction file, which will soon be fully compliant with the PSI-MI Causal Tab file format.

## 4 Results and discussions

SPV can be used to visualize signaling pathways and protein–protein interaction networks. It can display a variety of objects and connections and it offers essential export and on-the-fly filtering options.

It was conceived to be simple and straightforward. In fact, its integrations in any webpage only requires the following lines to be added in an HTML pages:

<script> baseurl = “.”; </script>

<script src=“./js/SPV_v1.0.js”></script>

The graph to visualize is a simple JSON object with elements representing links i.e.: source, target, interaction type and optionally score. Nodes and edges can respond to clicks displaying information in a list of key-values HTML texts.

Finally, to display the graph you only need to create a <div> block and call the main function as follows:

initGraph(links, node_labels, edge_labels, “graphArea”, 640, 480, “A”, 1, 0, 1);

The complexity of interactions in a pathway requires a rich variety of shapes and lines. The mTOR signaling pathway, as annotated in SIGNOR, is a good example of such complexity which, SPV makes it easier to understand and explore (http://signor.uniroma2.it/pathway_browser.php? organism=&pathway_list=SIGNOR-MS&x=20&y=14).

SPV is implemented in *mentha* to visualize protein-protein interactions (Calderone *et al.*, 2013), and in SIGNOR (Perfetto *et al.*, 2015) and DISNOR (Lo Surdo *et al.*, 2017) to draw casual relationships in signaling pathways.

## 5 Conclusions and future developments

Causal interactions are a relatively new focus of data curation and, as such, are still missing a clear web visualization tool. SPV is a JavaScript library for visualizing molecular interaction data with a particular focus on causal interactions.

SPV has been developed, tested and embedded in our major databases and we are planning to extend it to other online resources and possibly add other features to make data visualization even more effective without overlooking simplicity. In particular, we are focused on implementing other layouts and extend the SPV file format compatibility as new standards will become available.

We are releasing our code not only to let others benefit from our work but also to benefit from and promote open source collaborations in biological data visualization.
